# CD1d-independent NK1.1^+^ Treg cells are IL2-inducible Foxp3^+^ T cells co-expressing immunosuppressive and cytotoxic molecules

**DOI:** 10.3389/fimmu.2022.951592

**Published:** 2022-09-13

**Authors:** Hyun Jung Park, Sung Won Lee, Yun Hoo Park, Tae-Cheol Kim, Luc Van Kaer, Seokmann Hong

**Affiliations:** ^1^ Department of Integrative Bioscience and Biotechnology, Institute of Anticancer Medicine Development, Sejong University, Seoul, South Korea; ^2^ Department of Pathology, Microbiology and Immunology, Vanderbilt University School of Medicine, Nashville, TN, United States

**Keywords:** Treg cells, NK1.1, IFNγ, CD1d-independent NKT-like cells, IL2

## Abstract

Regulatory T cells (Treg) play pivotal roles in maintaining self-tolerance and preventing immunological diseases such as allergy and autoimmunity through their immunosuppressive properties. Although Treg cells are heterogeneous populations with distinct suppressive functions, expression of natural killer (NK) cell receptors (NKR) by these cells remains incompletely explored. Here we identified that a small population of Foxp3^+^CD4^+^ Treg cells in mice expresses the NK1.1 NKR. Furthermore, we found that rare NK1.1^+^ subpopulations among CD4^+^ Treg cells develop normally in the spleen but not the thymus through CD1d-independent pathways. Compared with NK1.1^-^ conventional Treg cells, these NK1.1^+^ Treg cells express elevated Treg cell phenotypic hallmarks, pro-inflammatory cytokines, and NK cell-related cytolytic mediators. Our results suggest that NK1.1^+^ Treg cells are phenotypically hybrid cells sharing functional properties of both NK and Treg cells. Interestingly, NK1.1^+^ Treg cells preferentially expanded in response to recombinant IL2 stimulation *in vitro*, consistent with their increased IL2Rαβ expression. Moreover, DO11.10 T cell receptor transgenic NK1.1^+^ Treg cells were expanded in an ovalbumin antigen-specific manner. In the context of lipopolysaccharide-induced systemic inflammation, NK1.1^+^ Treg cells downregulated immunosuppressive molecules but upregulated TNFα production, indicating their plastic adaptation towards a more pro-inflammatory rather than regulatory phenotype. Collectively, we propose that NK1.1^+^ Treg cells might play a unique role in controlling inflammatory immune responses such as infection and autoimmunity.

## Introduction

Immune responses must be tightly regulated in multiple layers, one of which is mediated by regulatory T (Treg) cells. Treg cells regulate various immune responses such as autoimmunity, hypersensitivity, infection, cancer, and organ transplantation (1–4). Treg cells are characterized by the expression of CD4, CD25 (IL2Rα) and transcription factor Foxp3 (forkhead box P3). In addition, these cells play a critical role in maintaining self-tolerance and immune homeostasis by regulating the effector functions of various immune cells such as T cells, dendritic cells (DCs), B cells, macrophages, natural killer (NK) cells, and natural killer T (NKT) cells (5, 6).

Based on their developmental locations, Treg cells are classified into two major subsets, thymus-derived Treg (tTreg) cells mediating central tolerance to self-antigens and peripheral Treg (pTreg) cells mediating peripheral tolerance to foreign antigens (7, 8). In addition, Treg cells can be called either natural Treg (nTreg) or inducible Treg (iTreg), depending on the inducibility of Foxp3 expression. For instance, nTreg cells express Foxp3 constitutively while iTreg cells are differentiated from naive conventional CD4^+^ T cells *via* transforming growth factor β (TGFβ)-mediated Foxp3 induction upon stimulation with antigen in the periphery (9). In addition, a few studies have demonstrated that TNFR2 signaling is also involved in inducing and maintaining the functions of Treg cells (10, 11). Furthermore, Treg cells have been subclassified into Th1-, Th2-, and Th17-types, depending on their distinct transcription factor profiles such as T-bet, GATA3, and RORγt, respectively (8). Treg cells exert immunosuppressive activity *via* multiple mechanisms: 1) the secretion of soluble anti-inflammatory factors (e.g., IL35, IL10, and TGFβ); 2) cell-to-cell contact-dependent suppression through inhibitory receptors (e.g., cytotoxic T-lymphocyte-associated protein 4 (CTLA4), glucocorticoid-induced TNFR family-related gene (GITR)); and 3) the release of cytolytic granules containing granzymes and perforin (12, 13).

Foxp3 (also known as scurfin) is a critically important transcription factor for controlling the development and functions of Treg cells (14). Although it is well known that Foxp3 protein is expressed in the classical CD4^+^ Treg cell population, several studies revealed that Foxp3-expressing populations are present in other cell types, including γδ T cells (15), CD8^+^ T cells (16), invariant NKT (iNKT) cells (17), and B cells (18) and these cells exert suppressive functions similar to classical CD4^+^ Treg cells.

iNKT cells are one of the innate-like T cells expressing a semi-invariant T cell receptor (Vα14Jα18 in mice and Vα24Jα18 in humans), and their development and activation are dependent on MHC I-like CD1d molecules (19, 20). Moreover, previous studies provide evidence that iNKT cells can express Foxp3 upon TGFβ and glycolipid antigen (e.g., α-GalCer) stimulation and that these cells acquire NK and Treg cell functions (17, 21). In addition to CD1d-dependent iNKT cells, CD1d-independent NKT-like cells exist that display a hybrid phenotype of NK and T cells (22). However, it remains unclear whether CD1d-independent NKT-like cells can become Treg cells. Thus, we characterized CD1d-independent NK1.1^+^ Treg cells that are not originated from iNKT cells. We demonstrated that these NK1.1^+^ Treg cells express Foxp3 and other Treg-related markers including CTLA4, GITR, and CD25.

## Materials and methods

### Study design

This study was designed to determine the phenotype of NK1.1^+^ Treg cells using Foxp3 green fluorescence protein (GFP) knockin (KI) reporter (hereafter Foxp3(GFP)) mice. To address this issue, splenocytes were harvested and further analyzed by flow cytometry. Furthermore, to explore CD1d dependency of NK1.1^+^ Treg cell development, we established Foxp3(GFP) CD1d knockout (KO) B6 mice and measured the NK1.1^+^ Treg cell population.

### Mice and reagents

Wild type (WT) C57BL/6 (B6) mice were purchased from Jung Ang Lab Animal Inc. (Seoul, Korea). CD1d KO B6 mice were provided by Dr. A. Bendelac (University of Chicago, IL, USA). Foxp3(GFP) WT B6 mice were obtained from Dr. Rho H. Seong (Seoul National University, Seoul, Korea). Foxp3(GFP) WT B6 mice were further crossed with CD1d KO B6 mice to obtain Foxp3(GFP) CD1d KO B6 mice. DO11.10 OVA-specific TCR transgenic (Tg) Balb/c × B6 F1 mice were generated by intercrossing DO11.10 Balb/c mice and B6 mice. All mice used in this study were maintained at Sejong University and used for experiments at 6-12 weeks of age. Mice were maintained on a 12-hour light/12-hour dark cycle in a temperature-controlled barrier facility with free access to food and water. Mice were fed a γ-irradiated sterile diet and provided with autoclaved tap water. Age- and sex-matched mice were used for all experiments. The animal experiments were approved by the Institutional Animal Care and Use Committee at Sejong University (protocol code SJ-20180804, approved on 4 August 2018). Lipopolysaccharide (LPS) derived from *Escherichia coli* (serotype 0111:B4) was purchased from Sigma-Aldrich (St. Louis, MO, USA). Ovalbumin (OVA) peptide_323–339_ (ISQAVHAAHAEINEAGR) was synthesized by Peptron Inc. (Daejeon, Korea).

### Cell isolation and culture

Splenic CD4^+^ T cells were isolated from B6 mice using a magnetically activated cell sorting (MACS) system (Miltenyi Biotec, Bergisch Gladbach, Germany), following the manufacturer’s instructions (23). CD4^+^ T cells were enriched >97% after MACS. Primary cells were cultured in RPMI 1640 (Gibco BRL, Gaithersburg, MD, USA) culture media supplemented with 10% FBS, 10mM HEPES, 2mM L-glutamine, 100 units/mL penicillin-streptomycin, and 5μM 2-mercaptoethanol. Total splenocytes (2 × 10^5^/well) were cultured with or without 5, 10, and 20 ng/ml of recombinant mouse IL2 (R&D Systems, Minneapolis, MN, USA).

### Calculation of NK1.1^+^ Treg cell number

Splenocytes were prepared after removing red blood cells (RBCs) using RBC lysis buffer and these cells were subsequently stained with trypan blue for counting viable cells under the microscope. After staining splenocytes with mAbs, the percentage of NK1.1^+^ Treg cells was evaluated by flow cytometry. The total cell number of NK1.1^+^ Treg cells was calculated by multiplying their percentage value with the splenocyte cell number. In addition, for experiments with CD4^+^ T cells purified using MACS, total splenocyte number (A) were counted under the microscope after trypan blue staining. Second, after staining splenocytes with mAbs, the percentage (B) of CD4^+^ T cells (CD3^+^CD4^+^) among total splenocytes and the percentage (C) of NK1.1^+^Foxp3^+^ cells among MACS-purified total CD4^+^ T cells were evaluated by flow cytometry. The total cell number of NK1.1^+^ Treg cells was calculated by multiplying the percentage value (B) of CD4^+^ T cells (CD3^+^CD4^+^) and percentage value (C) of NK1.1^+^Foxp3^+^ cells with the splenocyte cell number (A).

### Flow cytometry

The following monoclonal antibodies (mAbs) from BD Biosciences (San Jose, CA, USA) were used: Phycoerythrin (PE)- or allophycocyanin (APC)-conjugated anti-NK1.1 (clone PK-B6); fluorescein isothiocyanate (FITC)- or PE-Cy7-conjugated anti-TCRβ (clone H57-597); FITC-, PE-Cy7-, or APC-conjugated anti-CD3ϵ (clone 145-2C11); FITC-, PE-, or APC-conjugated anti-CD4 (clone RM4-5); PE- or APC-conjugated anti-CD25 (clone PC61); PE-conjugated anti-FasL (clone MFL3); PE-conjugated anti-CD152 (CTLA4) (clone UC10-4B9); PE-conjugated anti-NKG2D (clone C7); PE-conjugated anti-FR4 (clone FBP, FRd); PE-Cy7-conjugated anti-GITR (clone DTA-1); PE-conjugated anti-CD103 (clone M290); PE-conjugated anti-TNFα (clone MP6-XT22); and FITC- or PE-conjugated anti-IgG1 (isotype control) (clone R3-34). In addition, the following mAbs from Thermo Fisher Scientific were used: PE-Cy7-conjugated anti-KJ1-26 (clone KJ1-26); FITC-conjugated anti-Foxp3 (clone NRRF-30); PE-conjugated anti-IFNγ (clone XMG1.2); PE-conjugated anti-γc (clone TUGm2); PE-conjugated anti-IL4R (clone BVD6-24G2); PE-conjugated anti-IL2Rα (clone PC61); PE-conjugated anti-IL2Rβ (clone 5H4); PE-conjugated anti-IL15Rα (clone DNT15Ra); PE-conjugated anti-perforin (clone eBioOMAK-D); PE-conjugated anti-LAP (TGFβ) (clone TW7-16B4); and PE-conjugated anti-TRAIL (clone N2B2). To perform surface staining, cells were harvested and washed twice with cold 0.5% BSA-containing PBS (FACS buffer). To block Fc receptors, the cells were incubated with anti-CD16/CD32 mAbs on ice for 10 min and subsequently stained with fluorescently-labeled mAbs. Flow cytometric data were acquired using a FACSCalibur flow cytometer (Becton Dickson, San Jose, CA, USA) and analyzed using FlowJo software (Tree Star Inc., Ashland, OR, USA).

### Intracellular cytokine staining

For intracellular staining, splenocytes were incubated with brefeldin A, an intracellular protein transport inhibitor (10 μg/ml), in RPMI medium for 2 hrs at 37°C. The cells were stained for cell surface markers, fixed with 1% paraformaldehyde, washed once with cold FACS buffer, and permeabilized with 0.5% saponin. The permeabilized cells were then stained for an additional 30 min at room temperature with the indicated mAbs (PE-conjugated anti-IFNγ, anti-TNFα, anti-perforin, anti-CTLA4, anti-LAP (TGFβ), anti-IL10; or PE-conjugated isotype control rat IgG mAbs). Fixation and permeabilization were performed using a Foxp3 staining kit (eBioscience, San Diego, CA, USA) with the indicated mAbs (FITC-conjugated anti-Foxp3 or isotype control rat IgG mAbs). More than 5,000 cells per sample were acquired using a FACSCalibur, and the data were analyzed using the FlowJo software package (Tree Star, Ashland, OR, USA).

### CD1d/α-GalCer dimer staining for iNKT cells

To stain iNKT cells specifically, mCD1d/Ig fusion proteins (CD1d dimer; mouse CD1d dimerX, BD Biosciences, San Jose, CA, USA) were incubated overnight at 37°C with a 40-fold molar excess of α-GalCer (in PBS containing 0.5% Tween 20). The staining cocktail was prepared by mixing α-GalCer-loaded mCD1d/Ig proteins with FITC- or APC-conjugated anti-mouse IgG1 Ab (clone A85-1, BD PharMingen, San Diego, CA, USA) at a 1:2 ratio of dimer to anti-mouse IgG1 Ab. Subsequently, the mixture was incubated for 2 h at room temperature.

### Immunization protocols

OVA peptide_323–339_ (ISQAVHAAHAEINEAGR) was synthesized by Peptron Inc. (Daejeon, Korea). DO11.10 TCR Tg Balb/c × B6 F1 mice were immunized *via* subcutaneous injection with 20μg of the OVA peptide emulsified in complete Freund’s adjuvant (CFA) containing 5mg/mL of the heat-killed H37Ra strain of *Mycobacterium tuberculosis* (Difco Laboratories, Detroit, MI, USA) into the lower back. Ten days after immunization, these groups were sacrificed by CO_2_ inhalation for experiments.

### Statistical analysis

Statistical significance was determined using Excel (Microsoft, USA). Student’s t-test was performed for the comparison of two groups. *p < 0.05, **p < 0.01, and ***p < 0.001 were considered significant in the Student’s t-test. Two-way ANOVA analysis was carried out using the VassarStats (http://vassarstats.net/anova2u.html). #*p* < 0.05, ##*p* < 0.01, and ###*p* < 0.001 were considered to be significant in the two-way ANOVA.

## Results

### CD1d-independent NK1.1^+^ Foxp3^+^ cells are present in the spleen but not in the thymus

**Figure 1:**
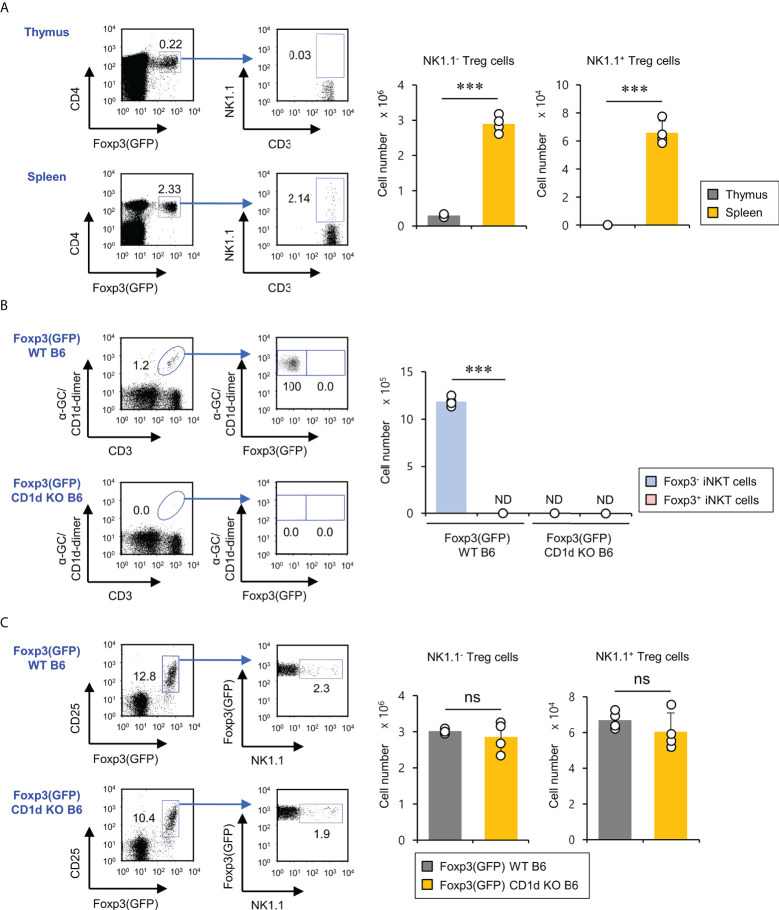
CD1d-independent NK1.1^+^ Treg cells exist in the spleen but not in the thymus. **(A)** Splenocytes and thymocytes were prepared from 8-week-old Foxp3(GFP) WT B6 mice. Left, the percentages of NK1.1^+^ subpopulations among splenic and thymic Treg cells (CD3^+^CD4^+^Foxp3(GFP)^+^) were plotted. Right, the absolute cell number of NK1.1^-^ Treg and NK1.1^+^ Treg cells was assessed by flow cytometry. **(B)** Left, the percentages of Foxp3(GFP)^+^ subpopulations among splenic iNKT cells (α-GC/CD1d dimer^+^CD3^+^) from 8-week-old Foxp3(GFP) WT or Foxp3(GFP) CD1d KO B6 mice were determined by flow cytometry. Right, the absolute cell numbers of splenic Foxp3^+^ iNKT or Foxp3^-^ iNKT cells from 8-week-old Foxp3(GFP) B6 or Foxp3(GFP) CD1d KO B6 were assessed by flow cytometry. **(C)** The percentages of NK1.1^+^ subpopulations among splenic Treg cells (CD3^+^CD4^+^Foxp3(GFP)^+^) (left) and the absolute cell number of NK1.1^-^ Treg and NK1.1^+^ Treg cells (right) from 8-week-old Foxp3(GFP) WT or Foxp3(GFP) CD1d KO B6 mice were determined by flow cytometry. The mean values ± SD (*n* = 4; per group in the experiment; Student’s t-test; ***p < 0.001) are shown. One representative experiment of three experiments is shown. ns, not significant; ND, not detected.

Previous studies have reported that treatment of human and murine CD1d-dependent iNKT cells with TGFβ and rapamycin can induce Foxp3 expression, leading to an increase in their suppressive capacities (17, 21). Thus, we wondered whether Foxp3-expressing NKT cells exist in the thymus and spleen under steady-state conditions. First, we examined NK1.1 expression of Foxp3^+^ cells in Foxp3(GFP) WT B6 mice. We found that NK1.1-expressing Foxp3^+^ cells were detected in the spleen (about 2% out of total Foxp3^+^ Treg cells) but not the thymus, implying that these cells might be pTreg cells rather than tTreg cells (**Figure 1A**). Moreover, we further confirmed the existence of these rare NK1.1^+^Foxp3^+^ Treg cells using isotype control mAb staining (**Figure S1**). Next, we decided to examine whether the developmental origin of these cells is the iNKT cell lineage. To address this issue, we generated Foxp3(GFP) CD1d KO B6 mice by crossing Foxp3(GFP) reporter B6 mice with CD1d KO B6 mice and subsequently evaluated the Foxp3 expression by iNKT cells. We confirmed that iNKT cells do not express Foxp3(GFP) during steady-state conditions. Interestingly, however, we found that both Foxp3(GFP) WT B6 and Foxp3(GFP) CD1d KO B6 mice had comparable numbers of NK1.1^+^ Treg cells expressing Foxp3(GFP), strongly indicating that NK1.1^+^ Treg cells do not derive from iNKT cells (**Figures 1B, C**). Thus, NK1.1^+^ Treg cells are not CD1d-restricted, although they are considered NKT-like cells expressing NK1.1 (**Figure S2**). Taken together, our findings suggest that NK1.1^+^ Treg cells develop in the peripheral tissue in a CD1d-independent manner.

### NK1.1^+^ Treg cells display a hybrid phenotype of Treg cells and NK cells

**Figure 2:**
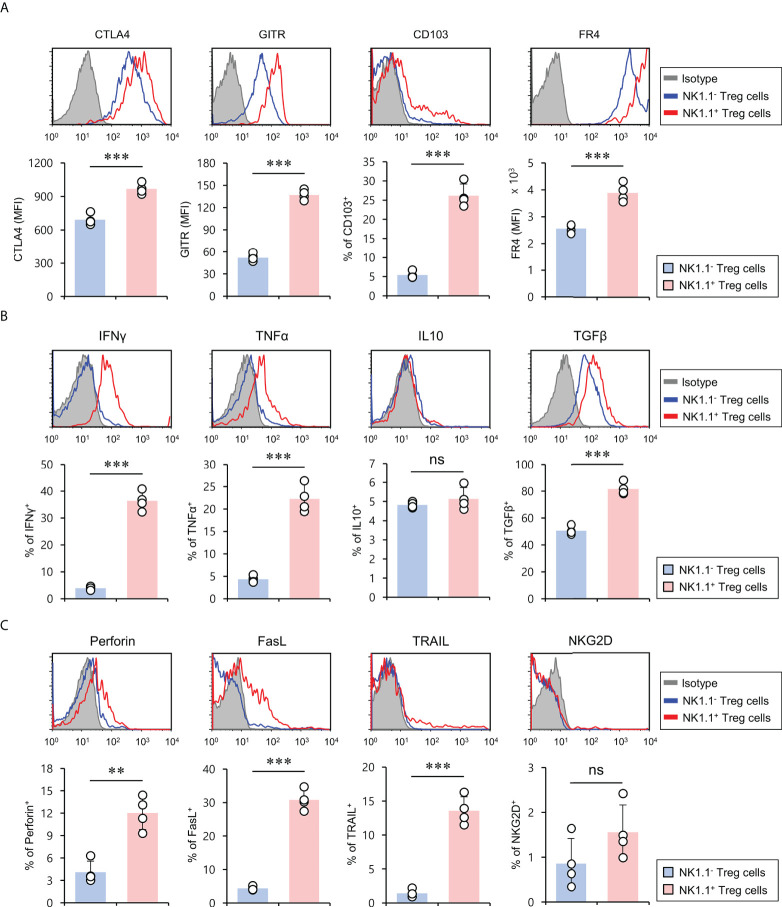
NK1.1^+^ Treg cells have the hybrid phenotype of Treg cells and NK cells. Splenic CD4^+^ T cells were isolated from 8-week-old CD1d KO B6 mice. **(A)** The expression of Treg cell-associated molecules (i.e., CTLA4, GITR, CD103, and FR4), **(B)** cytokines (i.e., IFNγ, TNFα, IL10, and TGFβ), and **(C)** NK cell-associated molecules (i.e., perforin, FasL, TRAIL, and NKG2D) on NK1.1^-^ Treg (NK1.1^-^CD4^+^Foxp3^+^) and NK1.1^+^ Treg (NK1.1^+^CD4^+^Foxp3^+^) cells were evaluated by flow cytometry. Upper, representative FACS histogram; lower, summary figures. The mean values ± SD (*n* = 4; per group in the experiment; Student’s t-test; **p < 0.01, ***p < 0.001) are shown. One representative experiment of two experiments is shown. ns, not significant.

Treg cell-related molecules (e.g., CTLA4, GITR, CD103, and FR4) are constitutively expressed by Treg cells and are essential for maintaining Foxp3^+^ Treg cells (24). In particular, it has been previously demonstrated that CD103 (αEβ7) is an established marker for activated Treg cells with effector memory phenotypes (25). Thus, we investigated whether NK1.1 expression affects Treg marker expression of Foxp3^+^ Treg cells. To explore this possibility, we compared CTLA4, GITR, CD103, and FR4 expression of either NK1.1^+^ or NK1.1^-^Foxp3^+^ Treg cells under normal conditions. Unexpectedly, we found that NK1.1^+^ Treg cells expressed markedly higher levels of Treg-related molecules than NK1.1^-^ Treg cells, indicating that NK1.1^+^ Treg cells might display enhanced suppressive effector functions (**Figure 2A**). Since NKT cells rapidly produce various cytokines that play critical roles in immune responses (20, 26), we examined expression profiles of either pro-inflammatory or anti-inflammatory cytokines of NK1.1^-^ and NK1.1^+^ Treg cells. Compared with NK1.1^-^ Treg cells, NK1.1^+^ Treg cells expressed higher levels of both pro-inflammatory (IFNγ and TNFα) and anti-inflammatory (TGFβ) cytokines. However, anti-inflammatory IL10 expression was not significantly different between NK1.1^-^ and NK1.1^+^ Treg cells (**Figure 2B**). Because NKT cells can produce cytolytic molecules and NK cell stimulatory receptors (27, 28), we investigated whether NK1.1^+^ Treg cells also display these properties. The expression of cytolytic molecules such as perforin, FasL, and TRAIL was markedly higher in NK1.1^+^ Treg cells than NK1.1^-^ Treg cells. In addition, most NK1.1^+^ Treg lacked NKG2D expression, similar to NK1.1^-^ Treg cells (**Figure 2C**). Our immune profiling results support the notion that NK1.1^+^ Treg cells are endowed with hybrid functional properties of both NK cells and Treg cells.

### IL2 induces the expansion of NK1.1^+^ Treg cells

**Figure 3:**
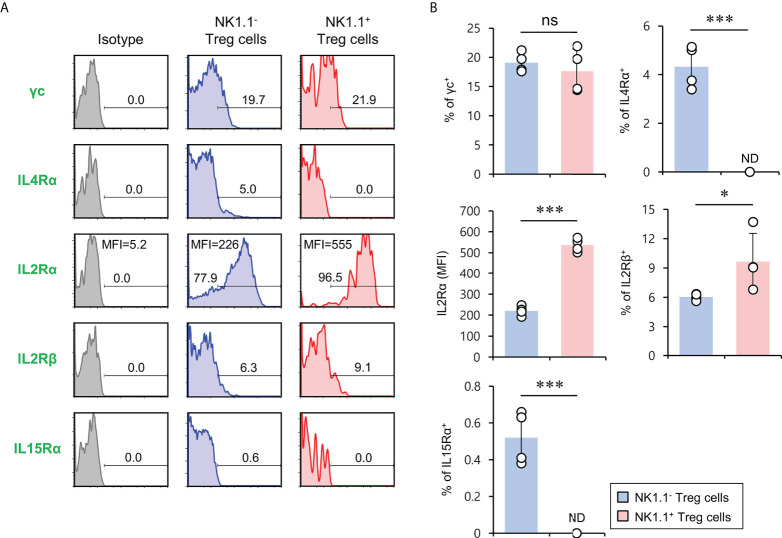
The expression of members of the common cytokine receptor γ-chain (γc) family of cytokine receptors on NK1.1^+^ Treg cells. Splenic CD4^+^ T cells were isolated from 8-week-old Foxp3(GFP) CD1d KO B6 mice. **(A, B)** Cell surface expression of the common cytokine receptor γ-chain (γc) family cytokine receptors (i.e., IL2Rγ, IL4Rα, IL2Rα, IL2Rβ, and IL15Rα) on NK1.1^-^ Treg (NK1.1^-^CD4^+^Foxp3(GFP)^+^) and NK1.1^+^ Treg (NK1.1^+^CD4^+^Foxp3(GFP)^+^) cells were evaluated by flow cytometry. **(A)** Representative FACS histogram; **(B)** summary figures. The mean values ± SD (*n* = 4; per group in the experiment; Student’s t-test; *p < 0.05, ***p < 0.001) are shown. One representative experiment of three experiments is shown. ns, not significant; ND, not detected.

**Figure 4:**
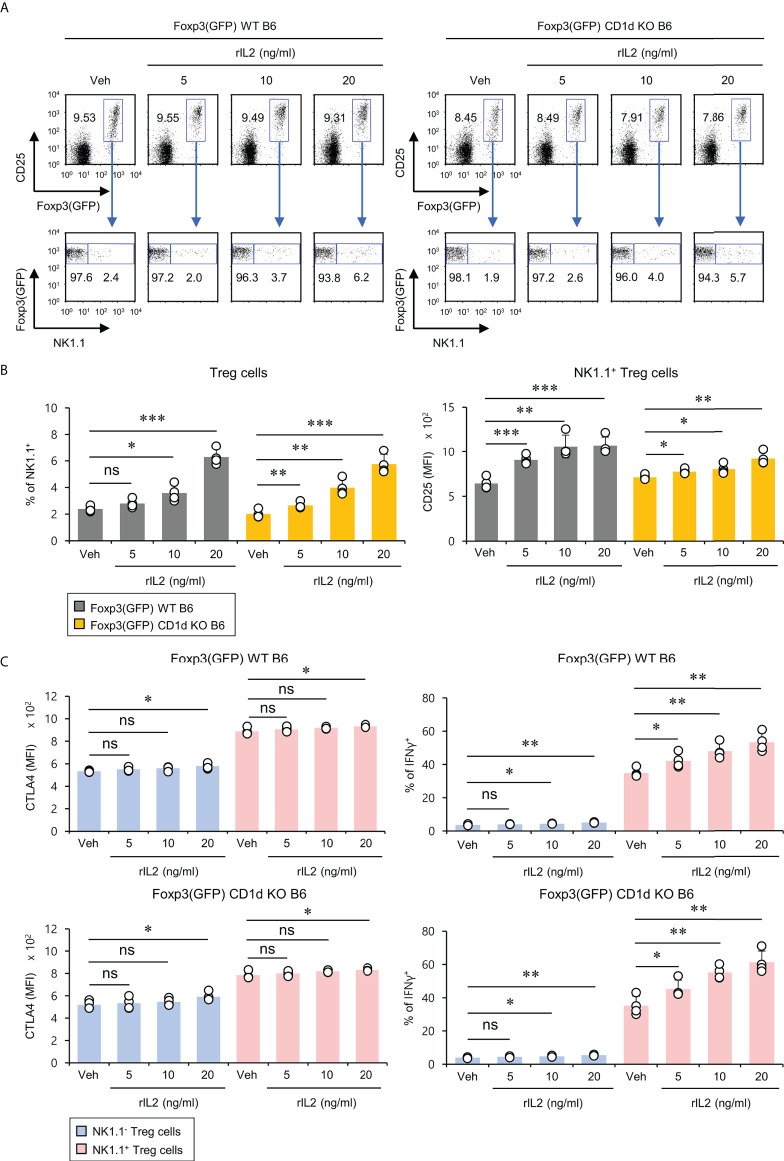
IL2 induces the expansion of NK1.1^+^ Treg cells. Splenocytes were prepared from 8-week-old Foxp3(GFP) WT and Foxp3(GFP) CD1d KO B6 mice. Subsequently, these cells were cultured with rIL2 (5, 10, or 20 ng/ml) for 5 days *in vitro*. **(A, B)** The frequency of NK1.1 and CD25 expression on NK1.1^-^ Treg (NK1.1^-^CD4^+^Foxp3(GFP)^+^) and NK1.1^+^ Treg (NK1.1^+^CD4^+^Foxp3(GFP)^+^) cells were determined by flow cytometry. **(C)** CTLA4 expression and IFNγ production by NK1.1^-^ Treg (NK1.1^-^CD4^+^Foxp3^+^) and NK1.1^+^ Treg (NK1.1^+^CD4^+^Foxp3^+^) cells were determined by flow cytometry. The mean values ± SD (*n* = 4 per group in the experiment; Student’s t-test; *p < 0.05, **p < 0.01, ***p < 0.001). One representative experiment of two experiments is shown. ns, not significant.

It has been reported that the signaling pathway of the common gamma chain (γc) cytokines (e.g., IL2, IL4, and IL15) influences the homeostasis and function of Treg cells in the periphery (29). Thus, to investigate whether the distinct phenotypes of NK1.1^+^ and NK1.1^-^ Treg cells are related to altered cytokine receptor (γc, IL4Rα, IL2Rα, IL2Rβ, and IL15Rα) expression, we compared their surface levels in these two populations. Although both NK1.1^+^ and NK1.1^-^ Treg cells expressed high levels of γc, NK1.1^+^ Treg cells expressed significantly higher levels of IL2Rα compared with NK1.1^-^ Treg cells. Furthermore, we found that NK1.1^+^ Treg cells displayed slightly but significantly higher levels of IL2Rβ expression than NK1.1^-^ Treg cells. However, IL4Rα and IL15Rα, which were only expressed by a small subset of NK1.1^-^ Tregs, were even lower on NK1.1^+^ Treg cells than NK1.1^-^ Treg cells (**Figures 3A, B**). Collectively, these results revealed that IL2 but not IL4 and IL15 cytokines might be closely associated with the differentiation of NK1.1^+^ Treg cells. Furthermore, it has been established that IL2 plays a pivotal role in the expansion and activation of NK cells and NKT cells (30–32) and in maintaining and developing Treg cells (33). Therefore, we considered that IL2, a well-known NK and Treg cell activator, participates in the expansion of NK1.1^+^ Treg cells expressing high levels of IL2R. To address this issue further, we examined immune responses of NK1.1^+^ Treg cells upon IL2 stimulation. We found that IL2 treatment significantly increased NK1.1 expression of Treg cells in a dose-dependent manner and greatly enhanced the expression of CD25 on NK1.1^+^ Treg cells, which was CD1d-independent (**Figures 4A, B**). In addition, IL2 stimulation significantly increased CTLA4 expression and IFNγ secretion in an iNKT cell-independent manner, suggesting that IL2 signaling contributes to inducing NK and Treg cells (**Figures 4C** and **S3**). Overall, these results provide strong evidence that IL2 signaling plays a pivotal role in expanding NK1.1^+^ Treg cells and inducing dual effector functions of NK and Treg cells in them.

### Expansion of antigen-specific NK1.1^+^ Treg cells

**Figure 5:**
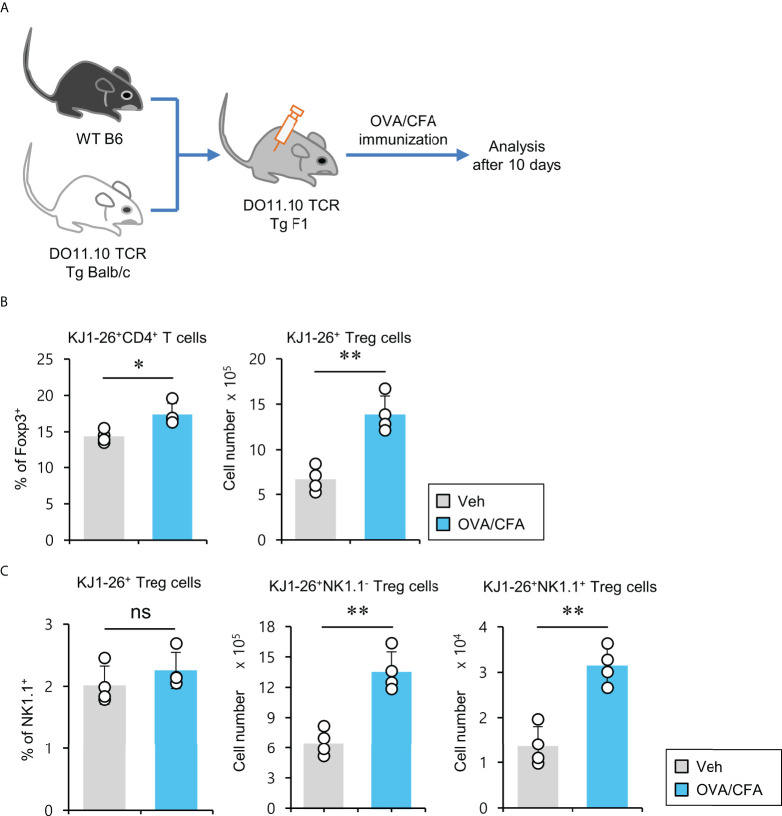
Expansion of antigen-specific NK1.1^+^ Treg cells after immunization with CFA/OVA **(A)** DO11.10 OVA-specific TCR transgenic (Tg) Balb/c × B6 F1 mice were immunized *via* subcutaneous injection with the OVA peptide emulsified in CFA. Ten days after immunization, splenic CD4^+^ T cells were enriched from these mice using MACS. **(B)** The percentage of Foxp3^+^ cells among splenic KJ1-26^+^CD4^+^ T cells and the absolute cell number of KJ1-26^+^ Treg cells were assessed by flow cytometry. **(C)** The percentage of NK1.1^+^ subpopulation among splenic KJ1-26^+^ Treg cells and the absolute cell numbers of both KJ1-26^+^NK1.1^-^ Treg and KJ1-26^+^NK1.1^+^ Treg cells were assessed by flow cytometry. The mean values ± SD (*n* = 4 per group in the experiment; Student’s t-test; *p < 0.05, **p < 0.01). One representative experiment of two experiments is shown. ns, not significant.

pTreg cells can be induced upon antigenic stimulation in the periphery (34). Thus, we wondered whether NK1.1^+^ Treg cells could be generated similarly to conventional pTreg cells. To address this issue, we employed ovalbumin (OVA)-specific DO11.10 TCR transgenic system and KJ1-26 mAb (specific for DO11.10 TCR-expressing T cells). Since the Balb/c strain does not express the NK1.1 marker, we used (Balb/c × B6) F1 mice to monitor NK1.1^+^ Treg cells expressing OVA-specific DO11.10 TCR and compared them with NK1.1^-^ conventional pTreg cells (**Figure 5A**). We found that OVA peptide-immunized mice displayed a significantly increased number of KJ1-26^+^CD4^+^ Treg cells (**Figure 5B**). Moreover, OVA stimulation significantly expanded both KJ1-26^+^NK1.1^-^ and KJ1-26^+^NK1.1^+^ Treg subpopulations compared with unimmunized mice (**Figure 5C**). Together, these results suggest that NK1.1^+^ Treg cells can be stimulated and expanded in an antigen-specific manner *in vivo*.

### LPS-induced systemic inflammation downregulates suppressive marker expression but upregulates TNFα production in NK1.1^+^ Treg cells

**Figure 6:**
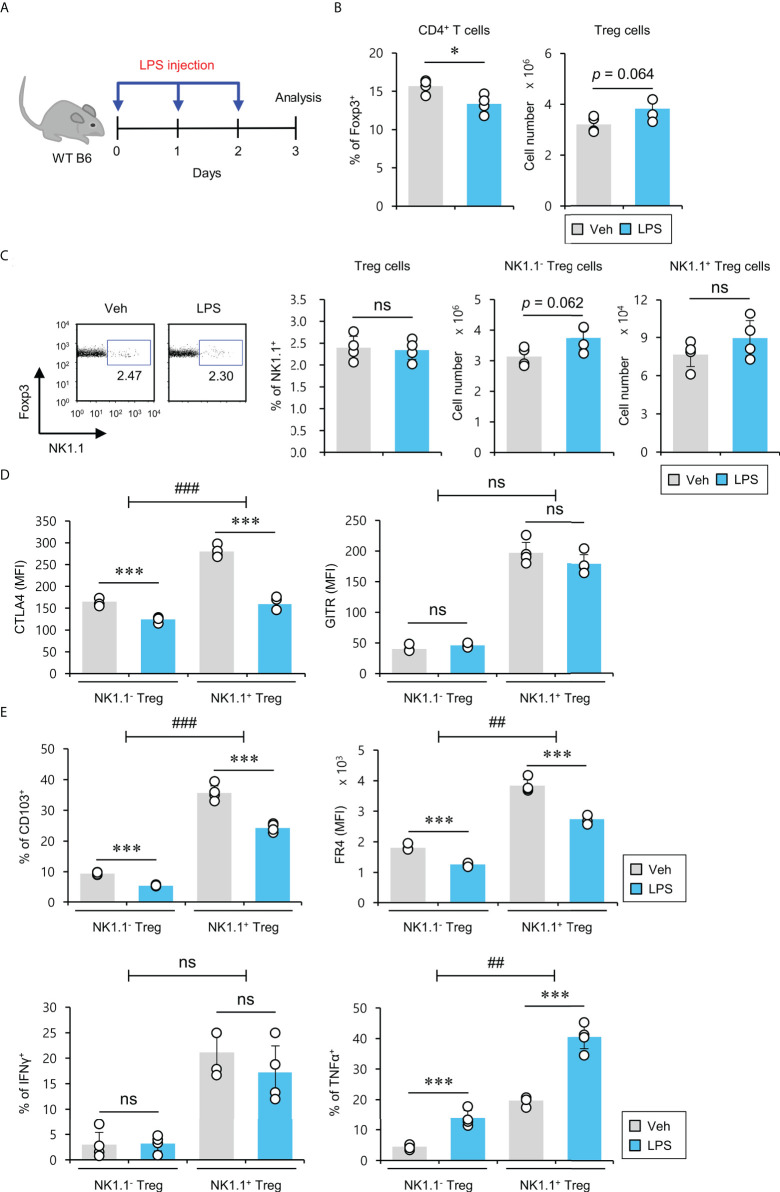
LPS-induced systemic inflammation downregulates immunosuppressive marker expression but upregulates TNFα production in NK1.1^+^ Treg cells. **(A)** WT B6 mice were i.p. injected either LPS (2 mg/kg) or vehicle (Veh) once per day for a total of 3 days, and total splenocytes from these mice were prepared one day after the last injection. **(B)** The percentage and absolute cell number of Treg cells were assessed by flow cytometry. **(C)** The percentage of NK1.1^+^ cells among splenic Treg cells and the absolute cell number of both NK1.1^-^ Treg cells and NK1.1^+^ Treg cells were assessed by flow cytometry. **(D, E)** The expression of Treg cell-associated molecules (i.e., CTLA4, GITR, CD103, and FR4) and cytokines (i.e., IFNγ and TNFα) on NK1.1^-^ Treg (NK1.1^-^CD4^+^Foxp3^+^) and NK1.1^+^ Treg (NK1.1^+^CD4^+^Foxp3^+^) cells were evaluated by flow cytometry. The mean values ± SD (*n* = 4 per group in the experiment; Student’s t-test; *p < 0.05, ***p < 0.001). One representative experiment of two experiments is shown. Two-way ANOVA (cell type × treatment) showed an interaction between these two factors (^##^ *p* < 0.01, ^###^ *p* < 0.001). ns, not significant.

Since it has been demonstrated that acute LPS-induced systemic inflammation limits the suppressive capacity of conventional Treg cells (35), we wondered if inflammatory stimulation can affect the differentiation and/or activation of NK1.1^+^ Treg cells. To explore this possibility, LPS was intraperitoneally (i.p.) injected into WT B6 mice once a day for a total of 3 days. First, we measured the frequencies and absolute cell numbers of both total and NK1.1^+^ Treg populations 24 hrs after the last injection. (**Figure 6A**). LPS treatment resulted in a modest but significant decrease in the frequency but not absolute cell number of splenic Treg cells. However, it did not significantly change the frequency and cell number of NK1.1^+^ Treg cells (**Figures 6B, C**). Next, we examined the expression of Treg cell-related molecules and pro-inflammatory cytokines in splenic NK1.1^+^ Treg cells. We found that NK1.1^+^ Treg cells from LPS-treated mice displayed a Treg phenotype with significantly decreased expression of CTLA4, CD103, FR4 but not GITR. In addition, LPS stimulation induced NK1.1^+^ Treg cells to increase TNFα but not IFNγ secretion (**Figures 6D, E**). Overall, these results provide strong evidence that LPS-induced systemic inflammation promotes the conversion of NK1.1^+^ Treg cells from a regulatory towards a pro-inflammatory phenotype, which indicates the potential plasticity of these regulatory NKT-like cells.

## Discussion

We have identified a small subset of CD4^+^ Treg cells expressing NK1.1 NKR (NK1.1^+^ Treg cells) in mice. Thymic Treg (tTreg) cells develop in the thymus through high-affinity TCR-MHC peptide interactions. As NK1.1^+^ Treg cells are not detectable in the thymus, our data demonstrate that these cells likely differentiate in the periphery. Previously, Monteiro *et al.* showed that Foxp3^+^ Vα14 TCR iNKT cells detected in the draining lymph node of the central nervous system could protect mice from experimental autoimmune encephalomyelitus by α-GalCer treatment. However, these cells were undetectable in the steady-state (17), which indicates that iNKT cell-derived Treg cells also differentiate in the periphery. Furthermore, although NK1.1^+^ Treg cells are a minor population (approximately 2% of total pTreg cells) in WT mice, they were unchanged in CD1d KO mice, indicating their CD1d-independence.

Treg cells maintain immunological tolerance and organ homeostasis. Specifically, pTregs participate in the control of immunity at sites of inflammation (36) and subsequently, these cells play an important role in repairing tissue damage after inflammation. Several cell types have been reported to facilitate the differentiation of pTreg cells. For example, tissue-resident macrophages in the lung (37) and CD103-expressing DCs in the lamina propria secrete retinoic acid through retinal dehydrogenase (RALDH) enzyme expression to promote Treg cell differentiation (38). In addition, mesenchymal stem cells (MSCs), a primary source for tissue regeneration, contribute to inducing Treg cell differentiation by secreting prostaglandin E2, IL10, and TGFβ (39–41). Recent studies have shown that MSCs can deliver various bioactive molecules (i.e., growth factors, cytokines, and microRNAs) *via* exosomes to neighboring immune cells for maintaining an anti-inflammatory environment (39, 42). As there is currently no information available on the cell types that can facilitate the differentiation of NK1.1^+^ Treg cells, it will be exciting to investigate the interaction between MSCs and NK1.1^+^ Treg cells during inflammation in future studies. Such information may be relevant to the development of immune therapies aimed at promoting tissue regeneration.

NK1.1^+^ Treg cells respond to recombinant IL2 treatment relatively better than NK1.1^-^ Treg cells. In addition, we found that NK1.1^+^ Treg cells increased upon antigen stimulation, indicating their antigen-specific proliferation. However, since pTreg cells can migrate from peripheral blood to tissues or vice versa under certain immunostimulatory conditions, the possibility of cell migration into the spleen rather than only proliferation cannot be completely ruled out (43). Therefore, examining the *in vivo* kinetics of NK1.1^+^ Treg cells upon antigen stimulation will be worthwhile in future studies.

IFNγ derived from NK cells is generally thought to play a pathogenic role in allograft rejection (44). In addition, NK cells activated by NKG2D ligands can cause lung ischemia-reperfusion injury, which occurs after lung transplantation (45). Moreover, MHC class I chain-related molecules (MICA and MICB) upregulated in allografts could trigger acute rejection through increased infiltration of NKG2D^+^NK1.1^+^ cells and CD8^+^ T cells into heart allografts (46). However, it has also been reported that IFNγ produced by allogenic Treg cells contributes to the prevention of graft-versus-host disease (GVHD) (47). Moreover, TNFα/TNFR2 signaling plays a pivotal role in Treg cell-mediated regulation of GVHD (10). Furthermore, inducible T-cell co-stimulator (ICOS) is indispensable for optimal survival and development of iTreg cells during chronic GVHD (48). Since NKG2D expression was not detectable on NK1.1^+^ Treg cells, which are capable of secreting high amounts of IFNγ and TNFα, it will be interesting to further investigate whether these cells play an essential role in the resolution of allograft rejection *via* TNFR2 and ICOS signaling-related mechanisms. Moreover, since NK1.1 is not expressed in Balb/c mice, alternative markers for NK1.1^+^ Treg cells applicable to all mouse strains will be essential to understanding the immunological significance of NK1.1^+^ Treg cells in the setting of mouse transplantation experiments such as GVHD. Pan-NK cell markers such as CD49a and DX5 can be tested first to determine whether they are expressed by NK1.1^+^ Treg cells (49). In addition, it will be intriguing to explore the putative human immune cells that may represent the counterpart to murine NK1.1^+^ Treg cells. A prior study identified Foxp3^+^ Treg cells expressing a characteristic human NK cell marker (i.e., CD56) in cancer tissues of hepatocellular carcinoma patients (50). In addition, previous studies have reported that a subpopulation of Treg cells expresses CD161 in humans, and these CD161^+^ Treg cells possess classic Treg signatures and pro-inflammatory phenotypes (51). Consistent with NK1.1^+^ Treg cells described in our study, human CD161^+^ Treg cells express significantly higher levels of Treg-associated molecules (e.g., CTLA4 and GITR) than CD161^-^ Treg cells in the blood of healthy adults (52). Based on these previous reports, it will be interesting to investigate whether CD56^+^ and CD161^+^ Treg cells might represent the human analogues to murine NK1.1^+^ Treg cells.

Previous studies demonstrated that Th1-type T-bet^+^ Treg cells develop in a STAT1-dependent manner and can produce IFNγ upon IL12 stimulation (53). Moreover, *in vitro* treatment with IL12 and TGFβ potentially promotes the generation of IFNγ^+^ Treg cells in the presence of IL2 (54). Although IL15 contributes to inducing NK1.1^+^ expression in CD8^+^ T cells (55) and enhancing Foxp3 expression in Treg cells (56), it is still unclear what factors can induce both NK markers (i.e., NK1.1) and Foxp3 in conventional CD4^+^ T cells. In future studies, it will be worthwhile to examine what signaling pathway can participate in NK1.1^+^ Treg cell differentiation.

Our results suggested the functional plasticity of NK1.1^+^ Treg cells with both regulatory and pro-inflammatory phenotypes under inflammatory conditions in mice. It has previously been shown that human NK-like Treg cells can alter their functions from pro-inflammatory to immunosuppressive phenotypes, indicating their functional adaptation depending on their microenvironment (50). Likewise, NK1.1^+^ Treg cells with dual phenotypes during steady-state conditions may potentially display biased phenotypes depending on the niche they belong to. For example, IFNγ^+^ NK1.1^+^ Treg cells might lose their pro-inflammatory functions in the immunosuppressive tumor microenvironment. In contrast, it has also been reported that IFNγ^+^ Treg cells induced by IL12/TGFβ effectively suppress inflammatory disease such as colitis (54). Thus, it will be exciting to investigate whether NK1.1^+^ Treg cells exhibit protective or pathogenic effects in acute and chronic inflammatory diseases.

It has been reported that LPS directly activates Treg cells, ultimately resulting in the inhibition of neutrophil inflammatory responses (57). However, another study showed that acute LPS-induced inflammation rapidly suppresses STAT5 signaling and proliferation of Treg cells (35). These discordant findings regarding the effects of LPS on Treg cell development and functions may suggest the need for temporal control of Treg cell function during infection and inflammation. For example, Treg cells should be inhibited during the early phase of infections (i.e., LPS stimulation) to maximize cytotoxic immune responses against infectious agents. After pathogens are eradicated (or immune responses have subsided), Treg cells come to the frontline to participate in tissue repair and wound healing. Thus, such a regulatory immune homeostatic mechanism might explain why NK1.1^+^ Treg cells downregulate suppressive molecules but upregulate pro-inflammatory molecules in response to acute LPS exposure. In addition, previous studies showing that the TNFα/TNFR2 signaling pathway is required to stabilize Treg cells (10, 11) can support our contention that the higher TNFα-producing characteristics of NK1.1^+^ Treg cells during inflammation may reflect a crucial role of autocrine TNFα/TNFR2 signaling pathway in maintaining the stability of NK1.1^+^ Treg cells.

Furthermore, depending on NK1.1 expression, Treg cells consist of two subpopulations with distinct roles. NK1.1^+^ Treg cells might exist to regulate the anti-inflammatory function of conventional Treg cells in an antigen-specific manner to promote immune homeostasis. In our previous studies, we showed that an increase in exogenous IFNγ signaling negatively regulates pTreg cell development by repressing Foxp3 expression in the presence of NKT cells and natural killer dendritic cells (NKDC) (28, 58). Since Th1-type effector molecules and transcription factors can inhibit pTreg generation (59, 60), NK1.1^+^ Treg cells with IFNγ-producing and cytotoxic properties might be excellent candidates to re-establish immune competence by inhibiting overt conventional pTreg cells. Several previous studies have reported that NK cell marker expression associates with IFNγ-producing and cytotoxic properties in DCs and γδ T cells (28, 61, 62), suggesting their immunoregulatory roles. Therefore, we propose that NK1.1^+^ Treg cells are antigen-specific regulators of pTreg cells, and it will be of interest to investigate this possibility in the future.

## Data availability statement

The original contributions presented in the study are included in the article/**Supplementary Material**. Further inquiries can be directed to the corresponding author.

## Ethics statement

The animal study was reviewed and approved by Institutional Animal Care and Use Committee (Sejong University).

## Author contributions

HP: study concept and design, acquisition of data, analysis and interpretation of data, drafting of the manuscript, statistical analysis, obtained funding. SL: study concept and design, acquisition of data, analysis and interpretation of data, drafting of the manuscript, statistical analysis, obtained funding. YP: acquisition of data, analysis and interpretation of data. T-CK: acquisition of data, analysis and interpretation of data. LVK: interpretation of data and drafting of the manuscript, review of the manuscript. SH: study concept and design, acquisition of data, analysis and interpretation of data, drafting manuscript, statistical analysis, obtained funding, administrative, and material study supervision. All authors contributed to the article and approved the submitted version.

## Funding

This work was supported by the Basic Science Research Program through the National Research Foundation of Korea (NRF) funded by the Ministry of Education (NRF-2021R1I1A1A01051465 to HP. NRF-2021R1I1A1A01054418 to SL. NRF-2019R1A2C1009926 and NRF-2022R1A2C1009590 to SH).

## Conflict of interest

LVK is a member of the scientific advisory board of Isu Abxis Co., Ltd. (South Korea).

The other authors declare that the research was conducted in the absence of any commercial or financial relationships that could be construed as a potential conflict of interest.

## Publisher’s note

All claims expressed in this article are solely those of the authors and do not necessarily represent those of their affiliated organizations, or those of the publisher, the editors and the reviewers. Any product that may be evaluated in this article, or claim that may be made by its manufacturer, is not guaranteed or endorsed by the publisher.
