# Supplementary material for: CD1d-independent NK1.1+ Treg cells are IL2-inducible Foxp3+ T cells co-expressing immunosuppressive and cytotoxic molecules
Source: Front Immunol. 2022 Sep 13;13:951592. doi: 10.3389/fimmu.2022.951592 (PMC9513232; doi:10.3389/fimmu.2022.951592)
Supplement: Supplementary file 1 [file DataSheet_1.docx]

**Supporting documents to:**

**CD1d-independent NK1.1^+^ Treg Cells are IL2-inducible Foxp3^+^ T Cells Co-expressing Immunosuppressive and Cytotoxic Molecules**

**This document includes:**

**-Supplementary figures 1-3**


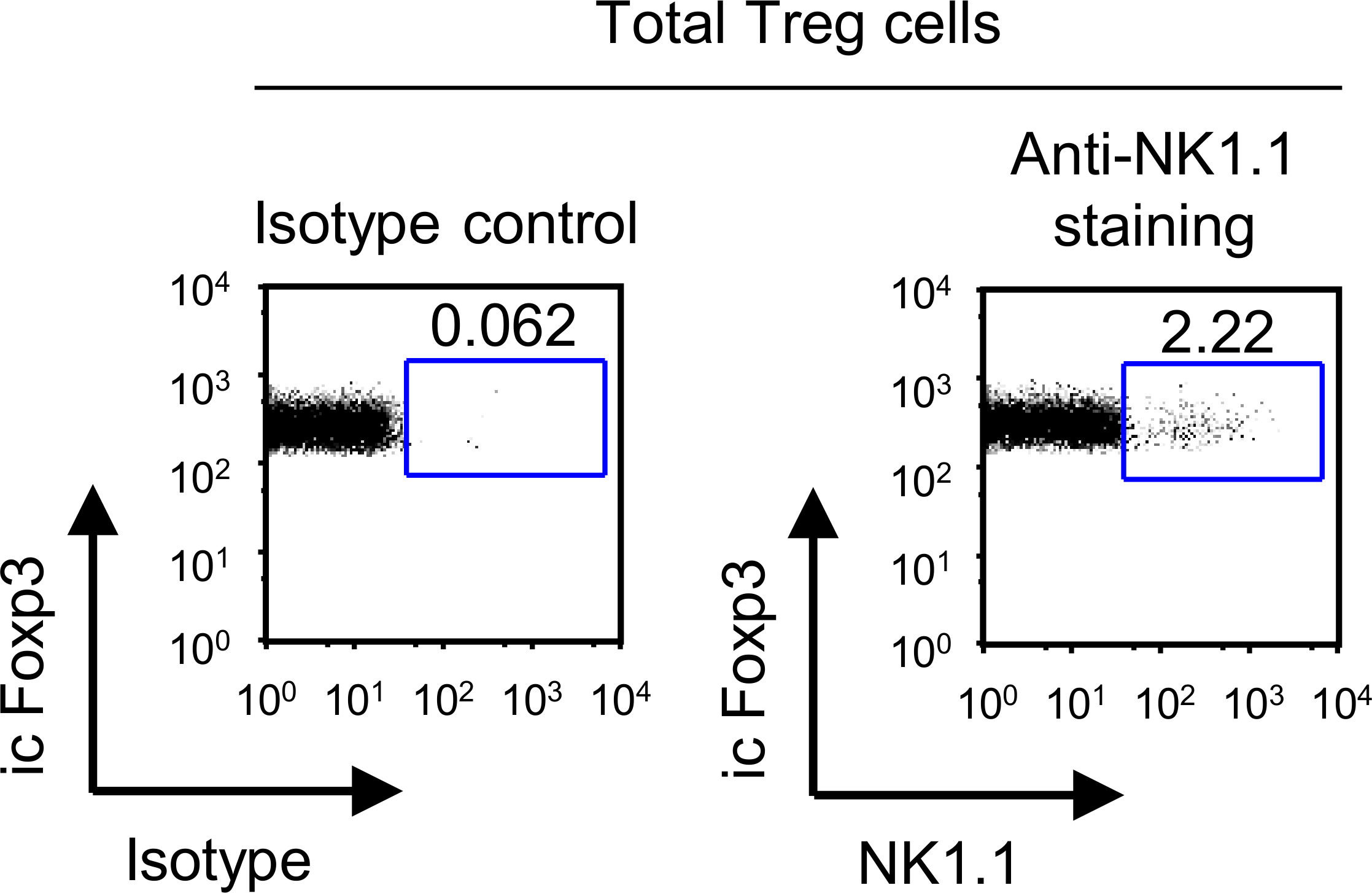


**Figure S1. Flow cytometric analysis of the NK1.1^+^ Treg cell population using isotype control mAb staining.**

Splenocytes were prepared from 8-week-old WT B6 mice. The percentages of NK1.1^+^ populations among total splenic Treg cells (CD3^+^CD4^+^Foxp3^+^) were determined by flow cytometry. An isotype control mAb was used for the anti-NK1.1 mAb to exclude any false positive background.


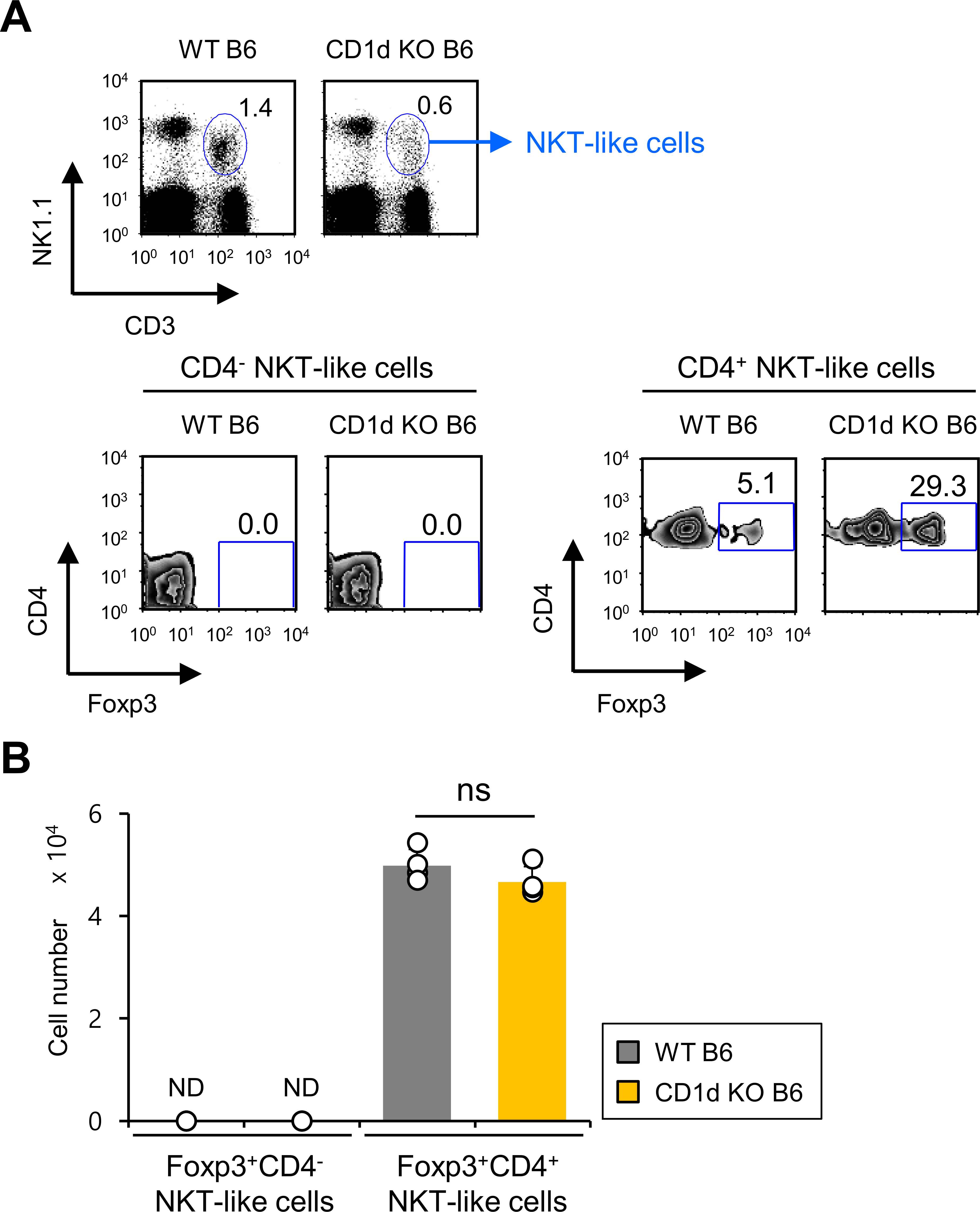


**Figure S2. Presence of splenic Foxp3^+^ NKT-like cells in CD1d KO B6 mice.**

(A) Splenocytes were prepared from 8-week-old CD1d KO B6 mice. Upper panel, the percentage of the NK1.1^+^CD3^+^ cell population among total splenocytes is plotted. Lower panel, the percentage of the Foxp3^+^ cell population among CD4^-^ and CD4^+^ NK1.1^+^CD3^+^ cells is plotted. (B) The absolute cell number of splenic Foxp3^+^CD4^-^ or Foxp3^+^CD4^+^ NK1.1^+^CD3^+^ cells was assessed by flow cytometry. The mean values ± SD (*n* = 4; per group in the experiment; Student’s t-test) are shown. One representative experiment of two experiments is shown.


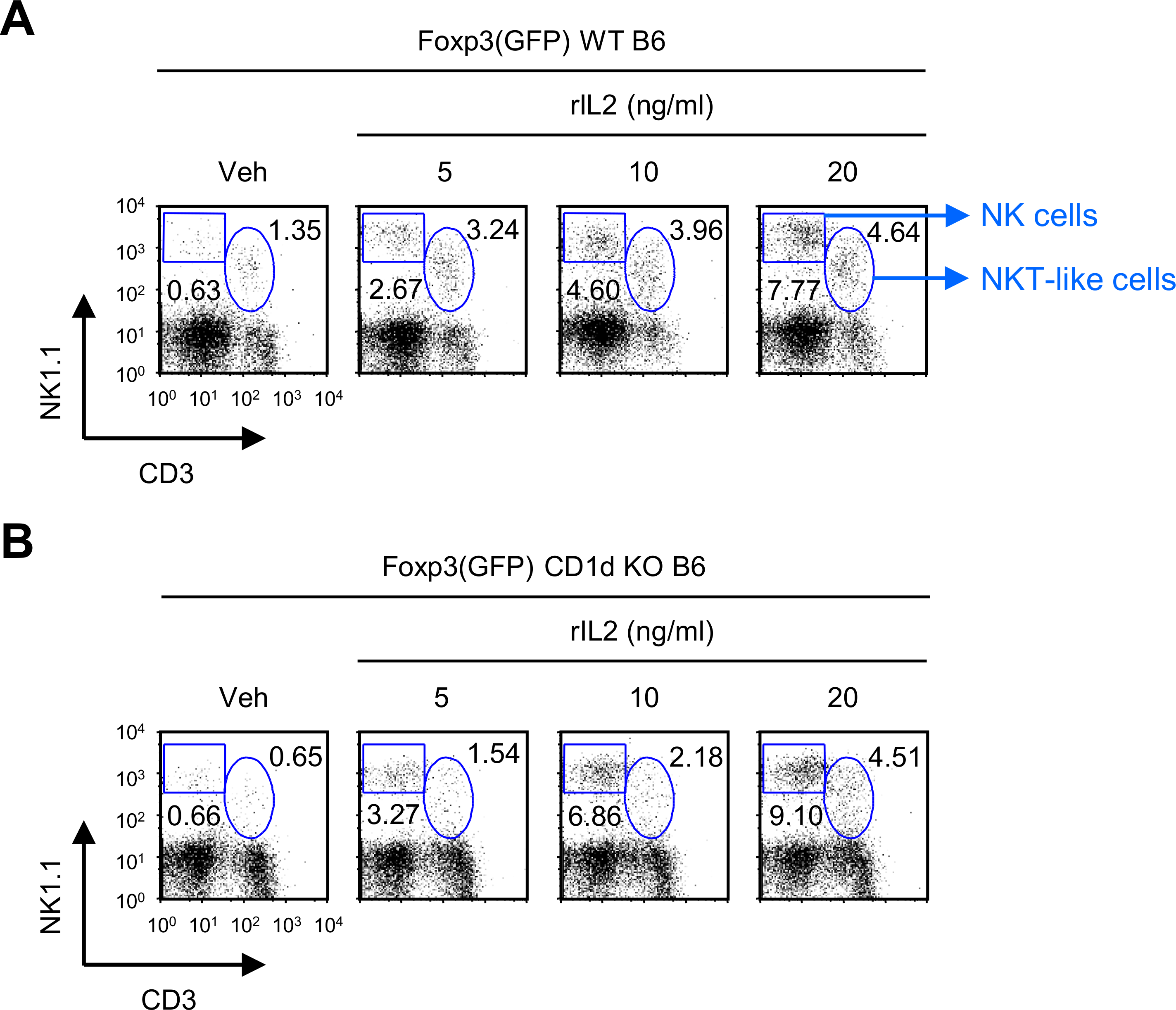


**Figure S3. IL2 induces the expansion of NK and NK1.1^+^CD3^+^ cells.**

(A-B) Splenocytes were prepared from 8-week-old Foxp3(GFP) WT and Foxp3(GFP) CD1d KO B6 mice. Subsequently, these cells were cultured with rIL2 (5, 10, or 20 ng/ml) for 5 days *in vitro*. The frequencies of NK cells (NK1.1^+^CD3^-^) and NKT and NKT-like cells (NK1.1^+^CD3^+^) were determined by flow cytometry. One representative experiment of two experiments is shown.
